# The Pattern of Unintentional Injuries and Poisoning Among Children Admitted to King Abdulaziz Medical City, Jeddah, From 2014 to 2018 in Saudi Arabia: A Cross-Sectional Study

**DOI:** 10.7759/cureus.30484

**Published:** 2022-10-19

**Authors:** Sara S Abed, Ethar Alboloshi, Jana Algithmi, Mashaer Alhusaini, Salwan Alsharif, Muhammad A Khan

**Affiliations:** 1 General Pediatric and Child Abuse, King Abdulaziz Medical City, Jeddah, SAU; 2 College of Medicine, King Saud Bin Abdulaziz University for Health Sciences, Jeddah, SAU; 3 College of Medicine, King Abdulaziz Medical City, Jeddah, SAU; 4 College of Medicine, King Abdullah International Medical Research Center, Jeddah, SAU

**Keywords:** unintentional injuries, types of injury, saudi arabia, pattern, poisoning, children

## Abstract

Background

Unintentional injuries and poisoning among children are prevalent and severe causes of hospitalization and impairment. The number of accidental injuries among children increases every year, leading to a huge burden on communities and health institutions.

Methods

This study is a retrospective analytical cross-sectional study. Charts from January 2014 to December 2018 were reviewed to estimate the epidemiological distribution and types of unintentional injuries among children aged 14 years or younger, including newborns, who were admitted to King Abdulaziz Medical City (KAMC), Jeddah, Saudi Arabia.

Results

In this study, 353 children were included. Patients were those admitted to King Abdulaziz Medical City (KAMC), Jeddah, due to unintentional injuries and whose age varies from birth to 14 years. Most of the injured children were males (60.1%), but interestingly more female patients suffered from fracture injuries than males. The most common injury was found to be falling (38.5%), followed by road traffic accidents (RTA) (26.1%). The frequency of different types of injuries varied among different age groups. Infants' injuries were mainly falling (50%), while RTA was the most common injury among adolescents (94.7%). Moreover, the head and neck area was the most affected site contributing 39.1% of all sites. Outcomes of the accidental injuries were assessed as full recovery, disability, or death.

Conclusion

This study showed the proportion of unintentional injuries among children from birth to 14 years old in KAMC, Jeddah, Saudi Arabia. Overall, the most prevalent type of injury found in our study was falling. Moreover, injuries were more common among male patients. We concluded that most injuries could be avoided if parents or children's guardians practiced protective strategies mainly by ensuring a safe and clear environment for the children.

## Introduction

Globally, traumatic unintentional injuries and poisoning among children are devastating causes of hospitalization and impairment [1]. Annually, 12,000 children die due to accidental traumas, and the number of children treated in an ED exceeds 9.2 million [2]. In Saudi Arabia, 44.7% of all cases that came to the hospital by the Saudi Red Crescent Authority (SRCA) in 2017 were caused by unintentional injuries [3]. According to the CDC, the patterns of accidental children's injuries may be one of the following: road traffic accidents (RTA), suffocation, falls, drowning, burns, and poisoning [2]. The type of injury that may affect children varies depending on their demographic characteristics, environment, and the types of activities they engage in [4]. In addition, socioeconomic class plays a significant role in jeopardizing children's safety, resulting in unintentional trauma [5]. 

Previous studies in Saudi Arabia showed the significance of unintentional injuries among children. However, there are no recent studies found covering 'Makkah's region. In 2013, a cross-sectional study in Makkah conducted an interview survey on 172 random mothers with children less than 12 years old attending the AlRusifah vaccination clinic. The study concluded that the prevalence of injuries was 20.9%, with falls as the leading type of injury [6]. Another retrospective study was conducted in King Abdulaziz Medical City in Riyadh, including data from hospitalized patients from 2009 to 2014 [7]. The sample size was 1762 patients, which showed that blunt trauma, burns, and penetrating objects are the most frequent patterns of injuries [7]. Furthermore, a recent cross-sectional study conducted in Riyadh city investigating unintentional injuries among children 12 years or younger studied the factors associated with unintentional injuries in pediatric clinics [8]. A total of 283 participants were enrolled, and the prevalence of unintentional injuries in one year was found to be 24.7%. Regarding the types of injuries, falls caused 62.9% of the cases, and burns caused 22.9%. The main factors correlating with unintentional injuries were found to be male gender, attending daycare, and careless parents [8]. Thus, implementing prevention plans and raising awareness regarding unintentional injury is necessary, thanks to its high impact on morbidity and mortality [9].

Unfortunately, there is a paucity of data regarding trauma incidence and prevalence, specifically for Saudi Arabian children [9,10]. Our search in the literature did not succeed in finding recent estimates, although the Makkah region, which includes Jeddah, has witnessed the highest number of accidental injuries by the SRCA [3]. Therefore, the current study was carried out to determine the pattern, types, and the most common form of childhood unintentional injuries and poisoning among the population aged from birth to 14 years old in King Abdulaziz Medical City, Jeddah, Saudi Arabia. Furthermore, the second objective was to assess the associated risk factors with unintentional injuries and poisoning among the target population.

## Materials and methods

Study design and settings

This study is analytical cross-sectional and was conducted in King Abdulaziz Medical City (KAMC), National Guard Hospital, located in the Western region of Saudi Arabia, Jeddah. Our study targeted inpatient pediatric medical and surgical wards, pediatric intensive care unit (PICU), and burns unit.

Identification of study participants

This study included female and male pediatric patients (≤14 years old) admitted to pediatric medical and surgical units, burns unit, and PICU from January 2014 to December 2018. Moreover, only those 14 years old or younger were included as this age group is considered pediatrics in Saudi Arabia, while those 15 years old or older are not seen by pediatricians. Numerous unintentional injuries included RTA, burns, suffocation, falls, drowning, and poisoning. Pediatric patients who attended the ED without admission, outpatient clinics, and oncology patients were excluded due to the unavailability of medical record coding. This study used a convenient sampling technique. The sample size was calculated with a CI of 95% and a margin of error of 0.05. The admitted pediatric population in the hospital was estimated to be 4000 patients from 2014 to 2018 and 800 in one year. Thus, the recommended sample size was 351 cases.

Data collection process

We used charts from the medical record department to obtain data from January 2014 to December 2018. Data were collected as soft copies, using the BESTCare system, and hard copies. The BESTCare system is an all-in-one system that integrates the outpatient, inpatient, ICU, ER, and operating room units. BESTCare aims to contain the patient's medical history through an electronic medical record that contains an integrated patient history from all medical departments through an electronic medical record. A data collection sheet was used to collect the data from the medical record department. Our primary objective was to identify the most prevalent types and subtypes of unintentional injuries among children (≤14 years old) and the most prevalent types among different genders and age groups in KAMC, Jeddah, Saudi Arabia. The quality of accessed data was ensured by unifying data entry for different cases between all those handling data collection. No major data was missing because most cases were documented similarly. However, some mentioned the mechanism of injury thoroughly, while others did not. Variables that were considered were demographic characteristics, date of admission, site, type of injury, and whether it resulted in chronic disability or morbidity. We considered the outcome of disability as any structural, functional, or mental impairment that leads to usual activity limitations and restriction of participation in normal daily activities and social interactions. Furthermore, participants were classified based on the type of injury: RTA, suffocation, falls, drowning, burns, and poisoning. In addition, injuries were classified based on affected sites which involved the head and neck, back, upper limb, lower limb, abdomen, and others.

Ethical considerations

Ethical approval was obtained prior to data collection from the Bioethics Committee at King Abdullah International Medical Research Center (approval no. H-01-R-005). Furthermore, patient data were secured and used for study purposes only.

Data analysis

Data analysis was carried out using IBM SPSS Statistics for MacOS, version 26 (IBM Corp., Armonk, NY, USA). Descriptive statistics were conducted to summarize the data. Qualitative variables were presented as frequency and percentage. For inferential statistics, the Chi-square test and Fisher's exact test were used as fit. A p-value of <0.05 was considered significant.

## Results

A total of 353 pediatric patients admitted due to unintentional injury were included in this study, and their acquired data were analyzed. Out of the total, 60.1% (n=212) of cases were male patients. In general, two age groups represented the majority of the study sample, namely, school-age (6-12 y/o) 29.7% (n=105) and toddlers 29.2% (n=103). Table 1 shows the demographic data of children with unintentional injuries.

**Table 1:** Demographic data of children.

Variables	Groups	Frequency (n)	Percentage (%)
Gender	Male	212	60.1
	Female	141	39.9
Age Groups	Infants	36	10.2
	Toddlers	103	29.2
	Preschool	90	25.5
	School-age	105	29.7
	Adolescents	19	5.4

Among all age groups, falls were the most observed mechanism of injury, 38.5% (n=136). The second most common type of injury was due to RTA accounting for 26.1% (n=92). Poisoning and burns were having nearly similar results 16.7% (n=59) and 16.4% (n=58), respectively, followed by drowning 1.4% (n=5) and suffocation 0.8% (n=3). Almost all children recovered from injuries and returned to their baseline health, 91.2% (n=322), and the rest suffered from severe injuries, which resulted in significant disability, 7.4% (n=26), or mortality 1.4% (n=5). Detailed data related to injury types and outcomes are illustrated in Table 2.

**Table 2:** Types, subtypes, and outcome of injury.

Type of injury	Subtypes	Frequency (n)	Percentage (%)
Road Traffic Accidents	Cars	64	68.5
	Motorcycle	14	15.7
	Pedestrian	14	15.7
	Total	92	100
Poisoning	Medications	30	50.8
	Chemicals	25	42.4
	Others	4	6.8
	Total	59	100
Burns	Scald	45	77.6
	Flame	10	17.2
	Others	3	5.2
	Total	58	100
Falls	Fracture	93	69.1
	Others	43	30.9
	Total	136	100
Drowning		5	1.4
Suffocation		3	0.8
Outcome	Normal	322	91.2
	Disability	26	7.4
	Mortality	5	1.4

As shown in Table 2, most RTAs happened while children were car passengers, 68.5% (n=64). The remaining accident cases, 31.4% (n=28), were equally distributed among motorcycle and pedestrian accidents, 15.7% (n=14) each. Another type of injury among children is poisoning, in which half of these events, 50.8% (n=30), were a result of medication ingestion. Detergent ingestion caused 42.4% (n=25) of the poisoning cases, and the remaining most minor percentages, 6.8% (n=4), resulted from ingestion of other poisonous agents. Scald burns were found to be the most prevalent subtype of burns contributing to 77.6% (n=45), followed by flame burns at 17.2% (n=10) and other different burn causes at 5.2% (n=3). In addition, falls incidents led to different outcomes. Noticeably, most falling cases were severe enough to cause bone fractures, 69.1% (n=93), while the remaining 30.9% (n=43) gave rise to different complications.

**Figure 1:**
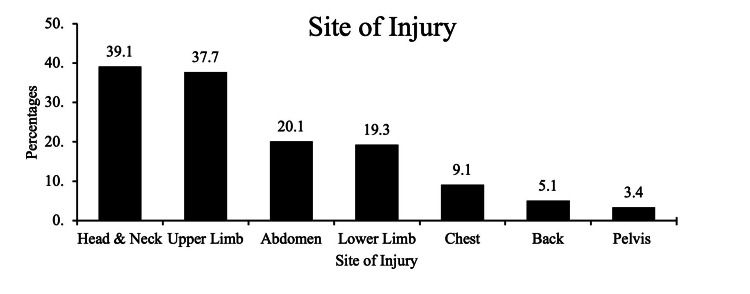
Sites of injury among all age groups (%).

As shown in Figure 1, the most affected site of injury was the head and neck area (39.1%), followed by the upper limb (37.7%), abdomen (20.1%), lower limb (19.3%), chest (9.1%), and the back area (5.1%). The pelvis area (3.4%) was the least observed injury site.

The association between age groups and injury types was investigated and found to be significant (p-value < 001) (Table 3). Half of the infants were admitted due to falling, 50% (n=18). Falling is also the most prevalent in both preschool, 45.6% (n=41) and school-age, 49.5% (n=52) groups. The commonest presentation for toddlers is poisoning, 34% (n=35) followed by burning, 29.1% (n=30). Almost all adolescents were admitted as cases of RTA, 94.7% (n=18), and only one case due to falls injury, 5.3% (n=1). 

**Table 3:** Distribution of injury types among different age groups. Fishers’ exact Test; P-value <0.001.

		Injury type						
		Falls N (%)	Drowning N (%)	Burns N (%)	Poisoning N (%)	Suffocation N (%)	RTA N (%)	Total (100%)
Age Groups	Infants	18 (50)	0	12 (33.3)	4 (11.1)	1 (2.8)	1 (2.8)	36
	Toddlers	24 (23.3)	3 (2.9)	30 (29.1)	35 (34)	1 (1)	10 (9.7)	103
	Preschool	41 (45.6)	2 (2.2)	9 (10)	15 (16.7)	1 (1.1)	22 (24.4)	90
	School-Age	52 (49.5)	0	7 (6.7)	5 (4.8)	0	41 (39)	105
	Adolescents	1 (5.3)	0	0	0	0	18 (94.7)	19
	Total	136 (38.5)	5 (1.4)	58 (16.4)	59 (16.4)	3 (0.8)	92 (26.1)	353

Additionally, the association between the gender of patients and the type/site of injury was studied. Interestingly, more female patients suffered from fracture injuries than male patients (p-value = 0.011). Moreover, several injury sites showed statistical significance with gender differences. For example, pelvic injuries were more common in female children than in their peer males, and this difference achieved statistical significance (p-value = 0.012). On the contrary, injuries in the head and neck area were more prevalent in male patients than in female cases (p-value = 0.024). Although other injury sites varied between male and female children, this variation failed to show statistical significance (Table 4).

**Table 4:** Bivariate analysis between gender and type/site. *Chi-square test; ** Fishers’ exact test.

Variable	Groups	Male	Female	P-value
Type of injury				0.011*
	Fracture injury	46 (49.46)	47 (50.54)	
	Other injuries	31 (72.09)	12 (27.91)	
Site of injury				
	Upper limb injury	72 (54.14)	61 (45.86)	0.077*
	No upper limb injury	140 (63.64)	80 (36.36)	
	Lower limb injury	45 (66.18)	23 (33.82)	0.252*
	No lower limb injury	167 (58.60)	118 (41.40)	
	Chest injury	20 (62.50)	12 (37.50)	0.767*
	No chest injury	192 (59.81)	129 (40.19)	
	Abdominal injury	44 (61.97)	27 (38.03)	0.712*
	No abdominal injury	168 (59.57)	114 (40.43)	
	Pelvic injury	3 (25.00)	9 (75.00)	0.012**
	No pelvic injury	209 (61.29)	132 (38.71)	
	Head and neck injuries	93 (67.39)	45 (32.61)	0.024*
	No head and neck injuries	119 (55.35)	96 (44.65)	
	Back injuries	12 (66.67)	6 (33.33)	0.557*
	No back injuries	200 (59.70)	135 (40.30)	

## Discussion

Unintentional injury is a devastating cause of hospitalization and morbidity among children worldwide [1]. A study by the world report about child unintentional injury prevention showed that accidental injuries are the most common cause of disability and mortality among pediatrics. Furthermore, the study results showed that unintentional injury prevalence is higher in the Eastern Mediterranean region than in the rest of the world [11]. Despite this high prevalence, there is insufficient data about accidental injuries among children in Saudi Arabia. Therefore, this study provided a recent estimate and described the pattern of unintentional injuries among pediatrics in King Abdulaziz Medical City, Jeddah, Saudi Arabia.

Among 353 pediatric patients, 60.1% were male, and 39.9% were female. The male predominance was also elicited in a global study that explored the pattern of unintentional injuries of 1559 children in four developing countries Bangladesh, Colombia, Egypt, and Pakistan [12]. The proportion of accidental injuries was higher among males, contributing to 65% of the cases consistent with the secondary analysis study of unintentional injuries, except for burns [11,12]. Also, male predominance was manifested in multiple local studies. One was conducted in Abha city, Saudi Arabia, and included children younger than 14 years old. Male children were more than female children in terms of unintentional injuries, 56.6% and 40.4%, respectively [8]. Similarly, another retrospective study was conducted in Riyadh, including children of the same age (14 y/o), which found that 68.4% of cases were males and 31.6% were females [7]. The higher proportion of unintentional injuries among male children in three different Saudi Arabia cities suggests that male patients are at higher risk than female children for several reasons. A possible explanation is that, locally, boys are more active and usually spend more time playing outside the house with their peers, unlike girls, who typically prefer to play inside. Another reason shared between both genders, especially at an age less than five, is curiosity which may result in burning and poisoning cases. Therefore, family education about children's close supervision should be an essential step in reducing and preventing morbidity and mortality resulting from unintentional injuries.

This study found that the most affected age groups were school-age (29.7%), followed by toddlers (29.2%). Likewise, a study conducted in Riyadh showed that unintentional injuries are more prevalent in the age group between 5 and 12 years (37.7%) [7]. Similarly, an international study of unintentional injuries found that 60% of the affected children were five years or older, and only 2% were younger than two years [12]. Thus, the school-age group is probably more susceptible to injuries due to their numerous exposures to different activities at home, school, and neighborhood.

The most commonly injured site was the head and neck area (39.1%), followed by the upper limb area (37.7%). Those results are consistent with a study conducted in Riyadh. They found 27.4% of injuries were in the head area and 13.7% in the right upper limb [7]. Falling was the most prevalent accidental injury affecting children (38.5%); this finding is consistent with other studies and documented by the WHO [7,12,13]. On the other hand, a study conducted in Riyadh found that RTAs were the most typical cause of unintentional injuries accounting for 52% [14]. Among all cases of unintentional falling, fractures were the most severe and main consequence (69.1%); this is similar to a multinational study conducted in four developing countries that discussed fractures and wounds as the serious outcome of falling [12]. Moreover, a local study conducted at King Khalid University in Riyadh revealed that most hand fractures in pediatrics were due to either falling or sports injuries [15]. None of the falling cases in the current study led to severe consequences such as paralysis. All the other consequences of falling were superficial bruises and lacerations with a good prognosis. Unfortunately, most of the falling cases occurred inside the home, which is supposed to be a safe environment for children; hence probably, parents are less cautious inside the home. However, falling outside the home can be anticipated and prevented. Therefore, it is essential to ensure a child-friendly environment that is safe and spacious to reduce fractures and falling risk.

The study showed that the second most common injury mechanism is RTAs, contributing to 26.1% of the cases. Similarly, a study conducted in Nepal showed that RTA (29.7%) is the most common injury after falling [16]. Another study found that the longest hospitalization stay was associated with RTA, leading to the highest morbidity and mortality rate, consistent with other studies [7,12]. In this study, the majority of RTA took place when patients were a passenger in cars (68.5%). Saudi Arabia is known for its high prevalence of car accidents, increasing the chances of unintentional injuries. Therefore, guardians should be aware that seat belts should be fastened, and young children should be seated in car seats. In contrast to this finding, another local study and a study of children in developing countries showed that most RTA occurred while the child was a pedestrian [12,17].

The analysis of poisoning cases showed that more than 50% of the poisoning resulted from medication ingestion followed by chemical ingestion. Likewise, a cross-sectional study conducted in King Khalid Hospital in Riyadh has congruent conclusions regarding the cause of poisoning in children [18]. Therefore, medication ingestion accidents are probably due to improper storage and not keeping medications out of children's reach. The present study found that burns represent 16.4% of all unintentional injury cases. Moreover, most burning cases are scald burns (77.6%), followed by flame burns (17.2%). Similar results were reported in another study in which scald burn accounted for 68.2%, followed by flame (23%) [19]. The high incidents of scald burn cases might be due to the improper handling of hot beverages such as tea, coffee, and milk. Therefore, a guardian's education is essential to reduce these injuries that may result in significant skin involvement requiring skin grafting surgery which may negatively impact children's social life and body image.

In this study, drowning was identified in only five cases (1.4%), three of which were males. The cases occurred only among toddlers and preschool age groups. Similarly, another paper conducted in Philadelphia studied drowning prevalence and found it to be low prevalence representing 4.9% of the total cases [20]. Most pediatric drowning cases are probably due to swimming without supervision or falling in pools without fences. Therefore, it is crucial to emphasize the importance of attentive adult supervision and protective pool fences to minimize drowning risks. Suffocation was the least type of injury affecting children accounting for merely three cases (0.8%). Moreover, all suffocation incidents were seen among male children and younger than five years old. Likewise, a study conducted in Oman found a similar prevalence of suffocation to this paper's findings [21]. The leading cause of choking was the ingestion of small foreign materials. Therefore, parents and guardians should always keep small objects out of children's reach.

Other recent studies whose findings are congruent with the findings of this study is a cross-sectional study conducted in Riyadh city on unintentional injuries among children 12 years old or above. The prevalence of injuries within a year was estimated to be 24.7%. This study concluded that injuries are most likely a result of accidental falls (62.9%), followed by burns (22.9%) [8]. Moreover, in 2021, a review by Albedewi H et al. included 36 studies that addressed a total of 20,136 children with injuries. This study identifies that falling was the leading cause of injury among Saudi children accounting for 31.9%, followed by motor vehicle accidents (25.1%). Furthermore, a total of 2,550 children were exposed to poisonous material. Most poisoning cases resulted from medication ingestion (39.9%), followed by toxic household products (25.7%). The rate of mortality was weighted to be 5.2% of all children with unintentional injuries. However, a higher rate was observed among critical cases, which include ICU burns (25.3%), head injuries (14.7%), and fractures (8.3%) [22]. According to the WHO, in 2016, at the beginning of the national transformation program, the fatality rate in Saudi Arabia that was caused by RTAs was as high as 28.8 compared to the general population, which is higher than both the low and high economic countries. It also had a substantial economic burden. This explains why RTAs are the second most common mechanism of injury in our study [23].

The limitations of our study are that it is a retrospective design and that children who presented to the ED without being admitted were not included due to the unavailability of medical record coding for this department. Thus, only inpatient data was accessed. Although our study focused on admitted patients, the current findings were consistent with other local and global studies that included patients who came to the ED and were not admitted. Another limitation is that the study was conducted in a single center. Thus, a multi-center study would reveal a better conclusion. Unfortunately, the patterns and risk factors of falling inside the home were not investigated in this study. Studying those factors can aid in formulating preventive strategies for falling inside the home. Also, studying the relationship between the admission length and the type of falling is essential in clustering the severe outcomes of falling.

## Conclusions

Unintentional traumatic injury among pediatrics is preventable yet a significant cause of morbidity and mortality. Generally, children are highly susceptible to getting injured due to their behavior and guardians' lack of education about the importance of supervision. This study aimed to identify the incidence and patterns of pediatric accidental injuries from birth to 14 years of age admitted to KAMC. The findings revealed that unintentional injuries are more prevalent in male children and school-age groups. In general, falling is the leading mechanism of accidental injury, followed by RTA. The commonest affected site of injury is the head and neck area, followed by the upper arm. Almost all patients recovered from the injuries, but few ended up with a permanent disability or death. The findings of the study highlight that unintentional injuries can be prevented by practicing cautious children's protection strategies and ensuring a safe environment. It is also advisable to raise awareness through public health campaigns about the importance of guardians' supervision, as this may significantly minimize the chances of accidental injuries. It is essential to apply a template in the health information system to ensure thorough documentation of accidental injuries. Proper documentation will serve as the cornerstone for future research, conducting primary prevention programs, initiating surveillance systems, and guiding legislators to set laws that help prevent the most common types of injury in our region. In addition, such data and contributions will, ultimately, aid in reducing risk factors, injuries, financial burdens, and complications caused by unintentional injuries. Moreover, it is crucial to have population-based studies that discuss the causes and associated factors of unintentional childhood injuries in Saudi Arabia and to have clear, organized prevention programs to reduce the number of motor vehicle accidents.

## References

[1] (2008). World Health Organization: World report on child injury prevention. https://www.who.int/publications/i/item/9789241563574.

[2] Borse NN, Gilchrist J, Dellinger AM, Rudd RA, Ballesteros MF, Sleet DA (2009). Unintentional childhood injuries in the United States: key findings from the CDC childhood injury report. J Safety Res.

[3] (2017). General Authority for Statistics, Kingdom of Saudi Arabia: Statistical Yearbook. https://www.stats.gov.sa/en.

[4] Sato N, Hagiwara Y, Ishikawa J, Akazawa K (2018). Association of socioeconomic factors and the risk for unintentional injuries among children in Japan: a cross-sectional study. BMJ Open.

[5] Gad A, AL-Eid R, Al-Ansary S, bin Saeed A, Kabbash A (2011). Pattern of injuries among children and adolescents in Riyadh, Saudi Arabia: a household survey. J Trop Pediatr.

[6] Aloufi LS (2017). Unintentional home injury in children up to age 12 years reported by mother attending vaccination clinic in Al-Rusifa PHC Center, Al-Mokarramah, Saudi Arabia (2013). Int J Med Res Prof.

[7] Alnasser A, Othman A, Mobaireek O, Alharthy N, Aljerian N, Al Zamel H (2018). Epidemiology of Pediatric Trauma at a Tertiary Hospital in Riyadh, Saudi Arabia. J Nat Sc Biol Med.

[8] Alkhamis KN, Abdulkader RS (2020). Assessment of unintentional childhood injuries and associated factors in the pediatric clinics of a tertiary care hospital in Riyadh, Saudi Arabia. J Family Community Med.

[9] (2012). National action plan for child injury prevention: an agenda to prevent injuries and promote the safety of children and adolescents in the United States. https://www.cdc.gov/safechild/pdf/national_action_plan_for_child_injury_prevention.pdf.

[10] (2004). World Health Organization: The global burden of disease. https://apps.who.int/iris/handle/10665/43942.

[11] Soori H, Khodakarim S (2016). Child unintentional injury prevention in Eastern Mediterranean Region. Int J Crit Illn Inj Sci.

[12] Hyder AA, Sugerman DE, Puvanachandra P (2009). Global childhood unintentional injury surveillance in four cities in developing countries: a pilot study. Bull World Health Organ.

[13] (2002). WHO: The injury chart book: a graphical overview of the global burden of injuries. https://www.who.int/publications/i/item/the-injury-chart-book-a-graphical-overview-of-the-global-burden-of-injuries.

[14] Alghnam S, Alkelya M, Al-Bedah K, Al-Enazi S (2014). Burden of traumatic injuries in Saudi Arabia: lessons from a major trauma registry in Riyadh, Saudi Arabia. Ann Saudi Med.

[15] Al-Jasser FS, Mandil AM, Al-Nafissi AM, Al-Ghamdi HA, Al-Qattan MM (2015). Epidemiology of pediatric hand fractures presenting to a university hospital in Central Saudi Arabia. Saudi Med J.

[16] Gupta PP, Malla GB, Bhandari R, Kalawar RP, Mandal M (2017). Patterns of injury and mortality in pediatric patients attending emergency department in a tertiary care center in Eastern Nepal. JNMA J Nepal Med Assoc.

[17] Crankson SJ (2006). Motor vehicle injuries in childhood: a hospital-based study in Saudi Arabia. Pediatr Surg Int.

[18] Alghadeer S, Alrohaimi M, Althiban A, Kalagi NA, Balkhi B, Khan AA (2018). The patterns of children poisoning cases in community teaching hospital in Riyadh, Saudi Arabia. Saudi Pharm J.

[19] el Danaf A, Alshlash S, Filobbos P, Rasmi M, Salem S (1991). Analysis of 105 patients admitted over a 2-year period to a modern burns unit in Saudi Arabia. Burns.

[20] Hwang V, Shofer FS, Durbin DR, Baren JM (2003). Prevalence of traumatic injuries in drowning and near drowning in children and adolescents. Arch Pediatr Adolesc Med.

[21] Al Rumhi A, Al Awisi H, Al Buwaiqi M, Al Rabaani S (2020). Home accidents among children: a retrospective study at a tertiary care center in Oman. Oman Med J.

[22] Albedewi H, Al-Saud N, Kashkary A, Al-Qunaibet A, AlBalawi SM, Alghnam S (2021). Epidemiology of childhood injuries in Saudi Arabia: a scoping review. BMC Pediatr.

[23] (2020). World Health Organization: Turning the tide against road traffic accidents. https://www.who.int/about/accountability/results/who-results-report-2020-mtr/country-story/2020/turning-the-tide-against-road-traffic-accidents.

